# Supplementary material for: Exploring the impact of the interthalamic adhesion on human cognition: insights from healthy subjects and thalamic stroke patients
Source: J Neurol. 2024 Jul 17;271(9):5985–96. doi: 10.1007/s00415-024-12566-z (PMC11377548; doi:10.1007/s00415-024-12566-z)
Supplement: Supplementary file 1 — Supplementary file1 (DOCX 1379 KB) [file 415_2024_12566_MOESM1_ESM.docx]

**Exploring the impact of the interthalamic adhesion on human cognition: insights from healthy subjects and thalamic stroke patients**

Julie P. Vidal^1,2^, Kévin Rachita^3^, Anaïs Servais^1^, Patrice Péran^2^, Jérémie Pariente^2,3^, Fabrice Bonneville^2,3^, Jean-François Albucher^2,3^, Lola Danet^2,3^, Emmanuel J. Barbeau^1^

*^1^CNRS, CerCo (Brain and Cognition Research Center) - Paul Sabatier University, Toulouse, France.*

*^2^INSERM, ToNiC (Toulouse NeuroImaging Center) – Paul Sabatier University, Toulouse, France.*

*^3^Purpan Hospital, Toulouse University Hospital Center, Neurology Department, Toulouse, France.*

Corresponding author: julie.vidal@univ-tlse3.fr

**Online Resources**


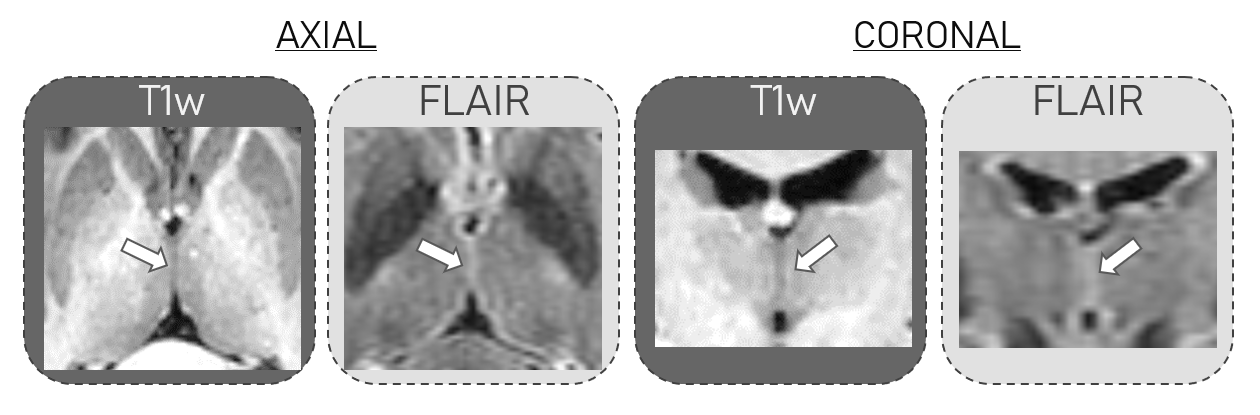


*Online Resource 1: Illustration of a kissing thalami case on the axial and coronal slice of a T1w (dark gray) or a FLAIR (light gray) image which does not allow identification of the presence or absence of the IA. Arrows indicate the contact between thalami where the IA could have been identified if present.*


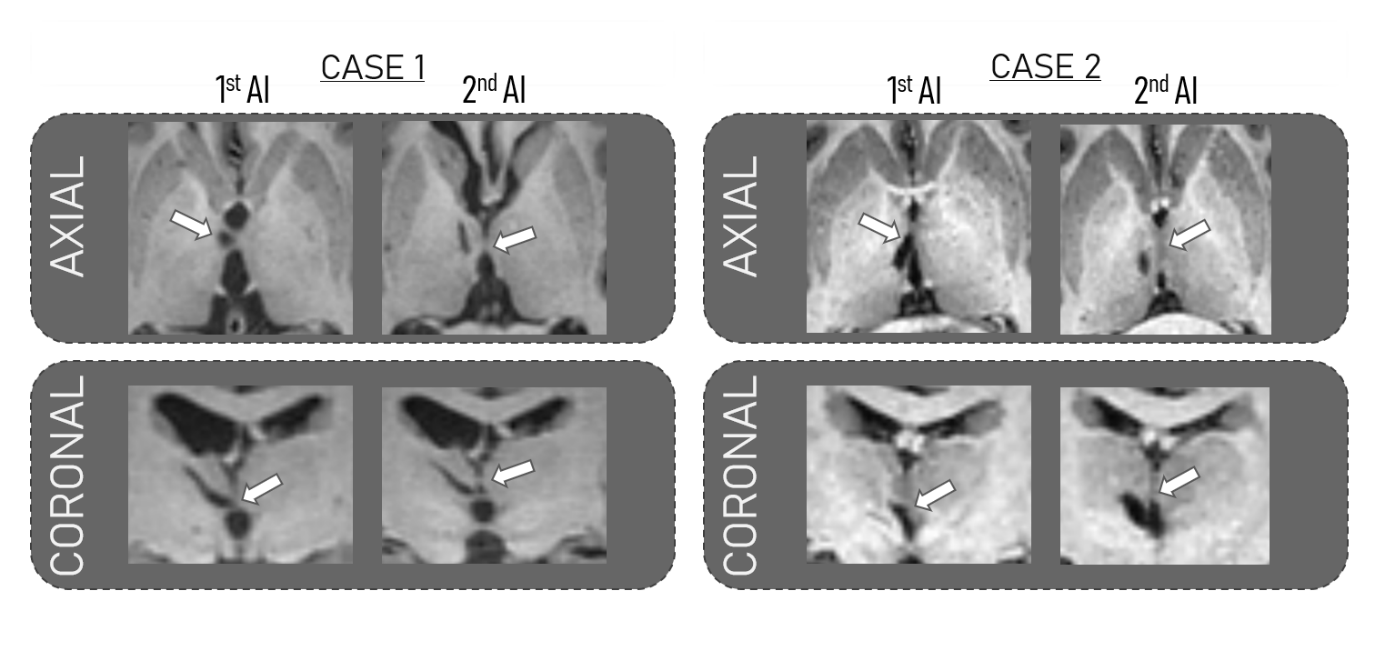


*Online Resource 2: Illustration of an axial and coronal slice of a T1w image from two patients with a double IA. The first image indicates the lesioned IA while the second demonstrates it is preserved. White arrows indicate the IA location.*


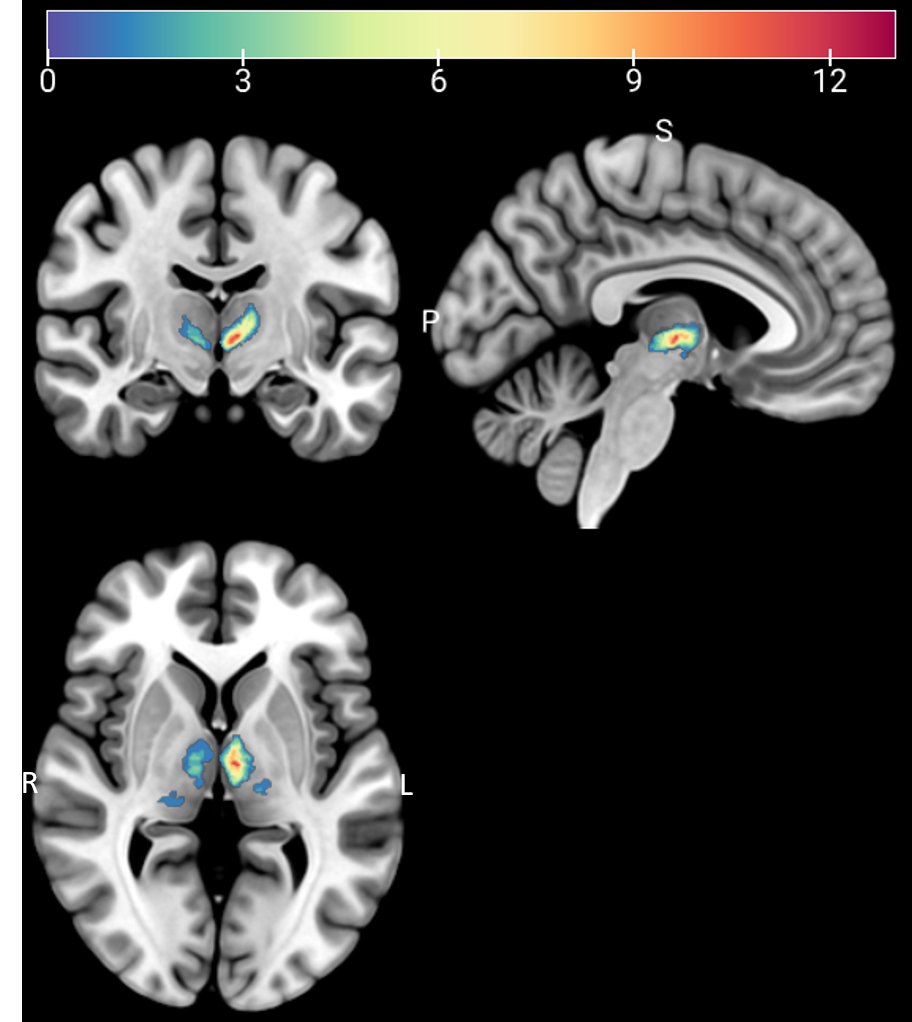


*Online Resource 3: Representation of all lesions from the 40 patients on the MNI152 template after normalization. The scale bar represents the number of overlapping lesions. L: Left; R: Right; S: Superior; P: Posterior.*
